# Visfatin Induces Senescence of Human Dental Pulp Cells

**DOI:** 10.3390/cells9010193

**Published:** 2020-01-12

**Authors:** Chang Youp Ok, Sera Park, Hye-Ock Jang, Takashi Takata, Moon-Kyoung Bae, Yong-Deok Kim, Mi Heon Ryu, Soo-Kyung Bae

**Affiliations:** 1 Department of Dental Pharmacology, BK21 PLUS Project, School of Dentistry, Pusan National University, Yangsan 50612, Korea; luriel@hanmail.net (C.Y.O.); sera8020@naver.com (S.P.); jho9612@pusan.ac.kr (H.-O.J.); 2 Periodontal Disease Signaling Network Research Center, School of Dentistry, Pusan National University, Yangsan 50612, Korea; mkbae@pusan.ac.kr (M.-K.B.); ydkimdds@pusan.ac.kr (Y.-D.K.); 3 Tokuyama University, Shunan, Yamaguchi 745-8566, Japan; ttakata@tokuyama-u.ac.jp; 4 Department of Oral Physiology, BK21 PLUS Project, School of Dentistry, Pusan National University, Yangsan 50612, Korea; 5 Dental and Life Science Institute, School of Dentistry, Pusan National University, Yangsan 50612, Korea; 6 Department of Oral and Maxillofacial Surgery, School of Dentistry, Pusan National University, Yangsan 50612, Korea; 7 Department of Oral Pathology, BK21 PLUS Project, School of Dentistry, Pusan National University, Yangsan 50612, Korea; apolllon@naver.com

**Keywords:** visfatin, senescence, dental pulp cells, telomere damage, SASP factors, NF-κB, inflammation

## Abstract

Dental pulp plays an important role in the health of teeth. The aging of teeth is strongly related to the senescence of dental pulp cells. A novel adipokine, visfatin, is closely associated with cellular senescence. However, little is known about the effect of visfatin on the senescence of human dental pulp cells (hDPCs). Here, it was found that in vivo visfatin levels in human dental pulp tissues increase with age and are upregulated in vitro in hDPCs during premature senescence activated by H_2_O_2_, suggesting a correlation between visfatin and senescence. In addition, visfatin knockdown by small interfering RNA led to the reduction in hDPC senescence; however, treatment with exogenous visfatin protein induced the senescence of hDPCs along with increased NADPH consumption, which was reversed by FK866, a chemical inhibitor of visfatin. Furthermore, visfatin-induced senescence was associated with both the induction of telomere damage and the upregulation of senescence-associated secretory phenotype (SASP) factors as well as NF-κB activation, which were all inhibited by FK866. Taken together, these results demonstrate, for the first time, that visfatin plays a pivotal role in hDPC senescence in association with telomere dysfunction and the induction of SASP factors.

## 1. Introduction

Dental pulp is a soft tissue that consists of the nerves, blood vessels, lymphatic vessels, connective tissues, and various types of cells within a tooth [1,2]. Dental pulp protects teeth against external invasive bacteria or irritants through its containment of various antibacterial substances, hormones, and immune cells, which prevents exogenous bacteria or substances from accumulating inside the dental pulp [1,3,4,5]. Advancing age or stressful conditions, such as inflammation, cause senescence of dental pulp cells, which leads to dental pulp aging and the diminished ability of teeth to protect themselves [6,7,8]. Therefore, it is important to understand the molecular mechanisms underlying the senescence of dental pulp cells and to develop a method to modulate these mechanisms to maintain healthy teeth.

Visfatin [pre-B-cell colony-enhancing factor (PBEF) or nicotinamide phosphoribosyltransferase (Nampt)] is an adipokine that is predominantly secreted but also exists in the nucleus and cytosol [9,10]. We and others have demonstrated that visfatin functions in neurite outgrowth, angiogenesis, tumorigenesis, and inflammation [11,12,13,14,15,16,17]. Studies in mice have shown that gingival visfatin expression is higher in obese versus lean mice and in older versus younger mice [18,19]. In addition, the levels of visfatin protein in saliva, serum, and tooth tissue in patients with periodontitis are much higher than those in healthy individuals [20,21,22,23,24]. Considering the increasing evidence of a connection between visfatin and inflammation [15,16,25], and that inflammation is a key driver of aging [26,27], visfatin is expected to contribute to cellular senescence and thereby, tissue aging. However, there are controversial results regarding the role of visfatin in cellular senescence. For example, it has been shown that visfatin reduces the senescence of endothelial progenitor cells, and its depletion promotes the senescence of retinal epithelial cells [28,29]. However, visfatin has also been shown to promote cellular senescence by inducing telomere damage in human vascular endothelial cells [30].

The role of visfatin in regulating the senescence of dental pulp cells is unknown. Therefore, the primary goal of this study was to evaluate the effect of visfatin on the senescence of human dental pulp cells. To achieve this, we assessed histological expression of visfatin in human dental pulp tissues of patients with different ages and in vitro effects of visfatin on human dental pulp cell line over a series of senescence markers including senescence-associated β-galactosidase, and expression of p21 and p53 proteins. We further aimed to reveal how visfatin affects the senescence of human dental pulp cells through molecular analysis such as NADPH consumption, telomere dysfunction, and senescence-associated secretory phenotype (SASP) genes expression.

## 2. Materials and Methods

### 2.1. Reagents

H_2_O_2_ was obtained from Junsei (Tokyo, Japan). Visfatin and FK866 were purchased from Adipogen (San Diego, CA, USA). Primary antibodies used in this study were as follows: rabbit anti-visfatin (Adipogen, San Diego, CA, USA), rabbit anti-p21 (Santa Cruz Biotech, Dallas, TX, USA), mouse monoclonal anti-p53 (Calbiochem, San Diego, CA, USA), rabbit anti-α-Tubulin (Bioworld, Minneapolis, MN, USA), mouse monoclonal anti-β-Actin (Abcam, Cambridge, MA, USA), mouse monoclonal anti-c-Myc (Santa Cruz Biotech, Dallas, TX, USA), mouse monoclonal anti-TRF-1 (Santa Cruz Biotech, Dallas, TX, USA), rabbit anti-phospho (Ser139)-histone H2AX (γH2AX) (Cell Signaling Technology, Danvers, MA, USA), and mouse monoclonal anti-NF-κB p65 (Santa Cruz Biotech, Dallas, TX, USA). Horseradish peroxidase-conjugated goat anti-rabbit and anti-mouse IgG were purchased from Thermo Fisher Scientific (Waltham, MA, USA). Alexa Fluor^®^ 488-conjugated goat anti-mouse IgG, Alexa Fluor^®^ 488-conjugated goat anti-rabbit IgG, Alexa Fluor^®^ 594-conjugated goat anti-mouse IgG, and Alexa Fluor^®^ 594-conjugated goat anti-rabbit IgG were purchased from Invitrogen (Camarillo, CA, USA).

### 2.2. Tissue Samples and Immunohistochemistry

Human dental pulp tissue specimens from surgically removed teeth were collected at the Department of Oral and Maxillofacial Surgery, School of Dentistry, Pusan National University, following the Pusan National University Dental Hospital Institutional Review Board (IRB) approval (PNUDH-2016-030) (Table 1). Formalin-fixed paraffin-embedded tissue samples were prepared, then 4 μm thick unstained sections were subjected to immunohistochemistry. After deparaffinization and blocking of endogenous peroxidase activity, antigen retrieval was performed using citric buffer solution, pH 6.0 (Invitrogen, Camarillo, CA, USA). According to the manufacturer’s instructions, the slides were boiled in citric buffer solution for 15 min and cooled for 25 min at 25 °C. Slides were incubated with the primary antibody against visfatin protein (1:100 dilution) at 4 °C overnight, followed by incubation with secondary antibody and DAB chromogen (SuperPictureTM 3rd Gen IHC Detection kit, Invitrogen, Camarillo, CA, USA). The slides were counterstained with Mayer’s hematoxylin and mounted. As a negative control, PBS solution was used instead of the visfatin antibody in the same IHC procedure. Our oral pathologist (M. H. R.), who was blind for the overall patient data and experimental design and performed immunohistochemistry, examined and evaluated the slides under light microscopy (Motic Instrument Inc., Hong Kong, China). Ten non-overlapping photographs (×200) were taken in each patient sample. For the evaluation, the cells with positively stained nuclei were counted in the pulp chamber and analyzed to determine differences between the young group (age range: 10–29 years old) and the older group (age range: 30–49 years old) using Statistical Package for the Social Sciences version 25.0 (SPSS Inc., Chicago, IL, USA).

### 2.3. Cell Culture

A cell line of immortalized human dental pulp cells (hDPCs) [31] were cultured in DMEM containing 10% fetal bovine serum (FBS; GibcoTM, Gaithersburg, MD, USA) and 1% antibiotics–antimycotics (GibcoTM, Gaithersburg, MD, USA). The cells were grown at 37 °C under a 5% CO_2_ atmosphere.

### 2.4. Gene Silencing by siRNA

A double-stranded siRNA oligonucleotide was designed and synthesized against visfatin (5′-CCACCCAACACAAGCAAAGUUUAUUTTdTdT-3′ and 3′-dTdTAAUAAACUUUGCUUGUGUUGGGUGG-5′), and a negative-control was purchased from Bioneer (Daejeon, South Korea). Oligofectamine (Invitrogen, Camarillo, CA, USA) was used as the transfection reagent, as recommended by the manufacturer. hDPCs were transfected with siRNA at 30% confluence for 4 h in minimal serum-free medium without antibiotics. Thereafter, growth medium containing thrice the normal concentration of serum was added without removing the transfection mixture, and cells grew for an additional 44 h until they reached confluency. Transient transfection with visfatin overexpression vector (pCI-Visfatin-Myc) was performed using X-tremeGENF HP DNA (Sigma Aldrich, St. Louis, MO, USA), according to the manufacturer’s instructions. Forty-eight hours after transfection, cells were fixed and subjected to immunocytochemistry.

### 2.5. RT-PCR

Total RNA was isolated from hDPCs using a Trizol reagent kit (Invitrogen, Camarillo, CA, USA). cDNA synthesis was performed using 1 μg of total RNA with MaximeRT premix (iNtRON Biotechnology, Sungnam, South Korea). The sequences of the oligonucleotide primers for the PCR were as follows: β-Actin, 5′-GACTACCTCATGAAGATG-3′ and 5′-GATCCACATCTGCTGGAA-3′; visfatin, 5′-GGATCCATGAATCCTGCGGCAGAAGC-3′ and 5′-CTCGAGATGATGTGCTGCTTCCAGTTC-3′; IL-1β 5′-GGATATGGAGCAACAAGTGG-3′ and 5′-ATGTACCAGTTGGGGAACTG-3′; IL-8 5′-ATGACTTCCAAGCTGGCCGTGGCT-3′ and 5′-CTCAGCCCTCTTCAAAAACTTCTC-3′; COX-2, 5′-TTCTTTGCCCAGCACTTCAC-3′ and 5′-CTGCTCATCACCCCATTCAC-3′.

### 2.6. Western Blot Analysis

Harvested cells were lysed in RIPA buffer (iNtRON Biotechnology, Sungnam, South Korea) containing a protease inhibitor cocktail (Roche, Mannheim, Germany). Protein extracts (30 μg/lane) were separated by SDS-PAGE and transferred to a nitrocellulose membrane (Amersham Pharmacia Biotech, Buckinghamshire, UK). The membrane was blocked with 5% skimmed milk in PBS containing 0.1% Tween 20 for 1 h at 25 °C and probed with appropriate antibodies. The signal was developed using the ECL detection system (Amersham Pharmacia Biotech, Buckinghamshire, UK).

### 2.7. SA-β-Galactosidase Staining Assay

The degree of SA-β-galactosidase activity was measured using a senescence assay kit (Senescence Cells Histochemical Staining; Sigma-Aldrich, St. Louis, MO, USA), according to the manufacturer’s protocol. In brief, hDPCs were treated with different concentrations of H_2_O_2_ to induce different degrees of cellular senescence. After 24 h, cells were washed twice in 1× PBS and fixed in 1× fixation buffer for 6–7 min at 25 °C, then washed in 1× PBS and incubated overnight with SA-β-galactosidase staining solution at 37 °C without CO_2_. Cells were photographed under a microscope (Olympus-IX71; Olympus, Toyko, Japan). Each experiment was performed in duplicate, and three separate experiments were carried out for each group.

### 2.8. NADP/NADPH Assay

NADP/NADPH levels were assessed using a colorimetric NADP/NADPH assay kit (Abcam, Cambridge, MA, USA) following the manufacturer’s instructions. Cells were lysed in an assay buffer provided in the kit. The lysates were deproteinized by passing through a 10 kD Spin column (Biovision, Milpitas, CA, USA). The assay was performed in a 24-well plate, and absorbance was measured with a Multimode Plate Reader Victor X3, P (Perkin Elmer, Hopkinton, MA, USA) at 450 nm.

### 2.9. Immunocytochemistry

Cells cultured on poly-L-lysine-coated coverslips were washed three times with PBS, fixed in 4% paraformaldehyde/PBS for 10 min at 25 °C, permeabilized with 0.01% Triton X-100 in PBS for 15 min at 25 °C, and then washed three times with PBS for 5 min. The cells were blocked with 1% BSA/0.3% Triton X-100/PBS for 1 h and labeled with the appropriate primary antibodies. After overnight incubation at 4 °C, the cells were incubated with Alexa Fluor 488-conjugated secondary antibodies for 1 h at 25 °C. Coverslips were mounted on slides with fluorescent mounting medium containing DAPI (Vector Laboratories, Burlingame, CA, USA). Cells were analyzed using a confocal microscope LSM 510 (Carl Zeiss, Oberkochen, Germany).

### 2.10. Statistical Analysis

a. The Analysis of Immunohistochemical Data

First, we performed Kolmogorov–Smirnov test to verify whether IHC data follow the normal distribution or not. Since IHC data was found not to be normally distributed, nonparametric statistical analysis such as Kruskal–Wallis test was performed. The ‘Young’ group consisted of patients aged from 10 years to 29 years, and patients aged from 30 years to 49 years were put together as ‘Old’ groups, which statistically analyzed the difference in number of visfatin-positive cells between these two groups. Statistical analysis was conducted using Statistical Package for the Social Sciences version 25.0 (SPSS Inc., Chicago, IL, USA) and was considered statistically significant if the p value is less than 0.05.

b. The Analysis of In Vitro Experiments

Data are expressed as the mean ± standard deviation (S.D.) obtained from at least three independent experiments. Statistical analysis was performed using Student’s t-test for data points and ANOVA for curves.

## 3. Results

### 3.1. Visfatin Levels Increase with Increasing Age in Human Dental Pulp

**Figure 1:**
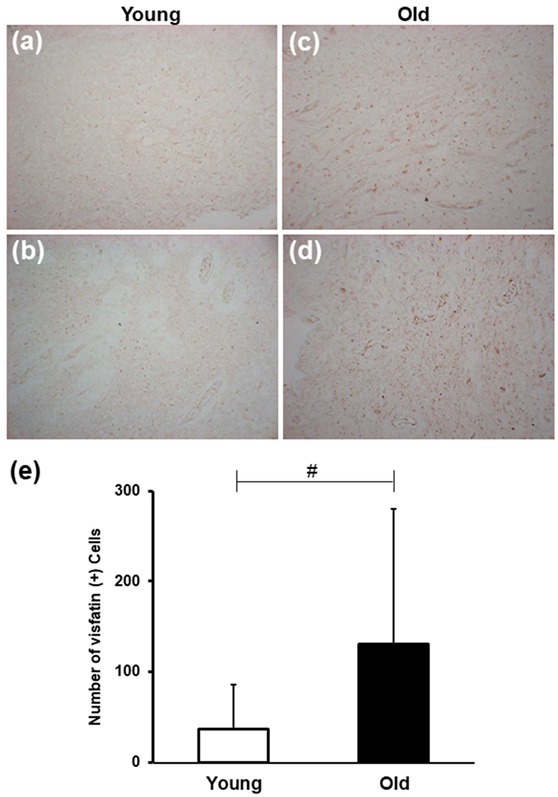
Age-induced changes in visfatin expression in dental pulp tissues. (**a**–**d**) Representative microphotographs of sections of dental pulp stained with anti-visfatin antibodies from individuals aged 17 (**a**), 20 (**b**), 35 (**c**), and 45 (**d**) years old. The number of visfatin-positive cells increased significantly in the Old group than in the Young group. All microphotographs were taken at an original magnification of × 200. Scale bar: 50 μm. (**e**) Bar graph illustrating the age-related changes in visfatin immunoexpression in the human dental pulp. Kruskal–Wallis test was performed to analyze the difference in number of visfatin-positive cells between Young group and Old group. # *p* < 0.0001.

To investigate whether visfatin expression changes with age, immunohistochemistry was performed using anti-visfatin antibodies on paraffin-embedded sections of human dental pulp tissues harvested at four different ages. While no, or very weak, visfatin staining was detected in samples from young individuals; its expression increased with increasing age (Figure 1a,b vs. Figure 1c,d). Quantification of the results showed a significant increase in visfatin levels with age (Figure 1e).

### 3.2. Visfatin Expression is Upregulated in Premature Senescent Dental Pulp Cells

**Figure 2:**
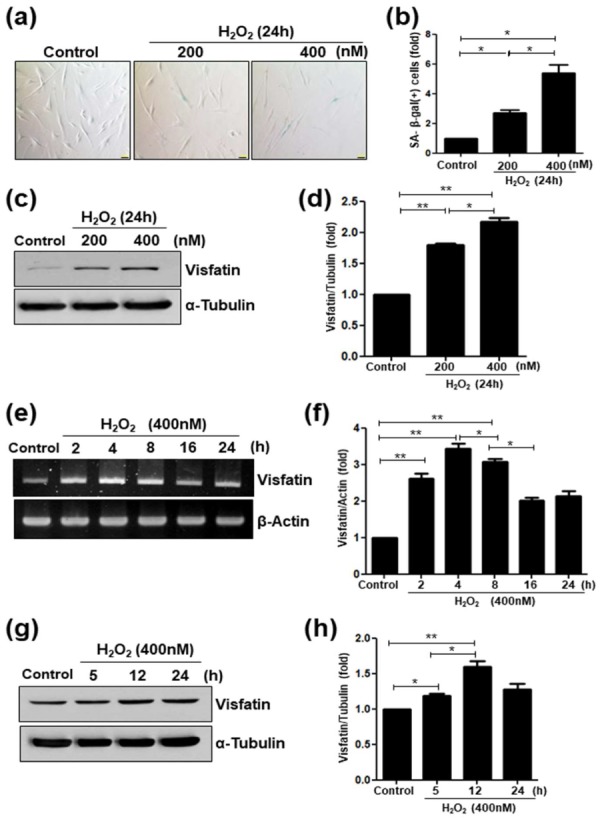
Upregulation of visfatin in H_2_O_2_-induced senescence of human dental pulp cells (hDPCs). (**a**–**d**) hDPCs were stimulated with different concentrations of H_2_O_2_ (0, 200, and 400 nM) for 24 h. (**a**) The cells were stained for the detection of the activity of senescence-associated (SA)-β-galactosidase. Scale bar: 200 μm. (**b**) Quantitative results for the percentage of SA-β-galactosidase positively stained cells. (**c**) Cells were treated with H_2_O_2_ (200 and 400 nM) for 24 h. Cell lysates were subjected to Western blotting for detecting the levels of visfatin or α-Tubulin used as the loading control. (**d**) Relative visfatin protein levels normalized with α-Tubulin protein levels. (**e**–**h**) Cells were incubated with H_2_O_2_ (400 nM) for different time periods (0–24 h). (**e**) Cell lysates were subjected to RT-PCR for determining visfatin mRNA expression. β-Actin was used as an internal control. (**f**) Relative visfatin mRNA levels normalized with the levels of β-Actin mRNA. (**g**) Cell lysates were subjected to Western blotting to detect the levels of visfatin protein. α-Tubulin was used as the loading control. (**h**) Relative visfatin protein levels were normalized with the levels of α-Tubulin protein. * *p* <0.1, ** *p* < 0.01.

H_2_O_2_ is an oxdative stress-inducing substance that causes the premature senescence of various types of cells [32]. To examine the effect of H_2_O_2_ on dental pulp cells, hDPCs were treated for 24 h with different concentrations of H_2_O_2_, then SA-β-galactosidase staining was assayed. H_2_O_2_ treatment enhanced the percentage of cells expressing SA-β-galactosidase (SA-β-gal), a marker of cellular senescence (Figure 2a,b), which is consistent with our previous study [33]. Next, the expression pattern of visfatin following H_2_O_2_-induced premature senescence was evaluated. H_2_O_2_ treatment in a concentration range of 200–400 nm increased visfatin protein levels in hDPCs in a dose-dependent manner (Figure 2c,d). In addition, the levels of visfatin mRNA and protein were increased in a time-dependent manner and peaked at 4 (Figure 2e,f) and 12 h (Figure 2g,h) after 400 nM H_2_O_2_ treatment, respectively.

### 3.3. Visfatin Silencing Delays Cellular Senescence

**Figure 3:**
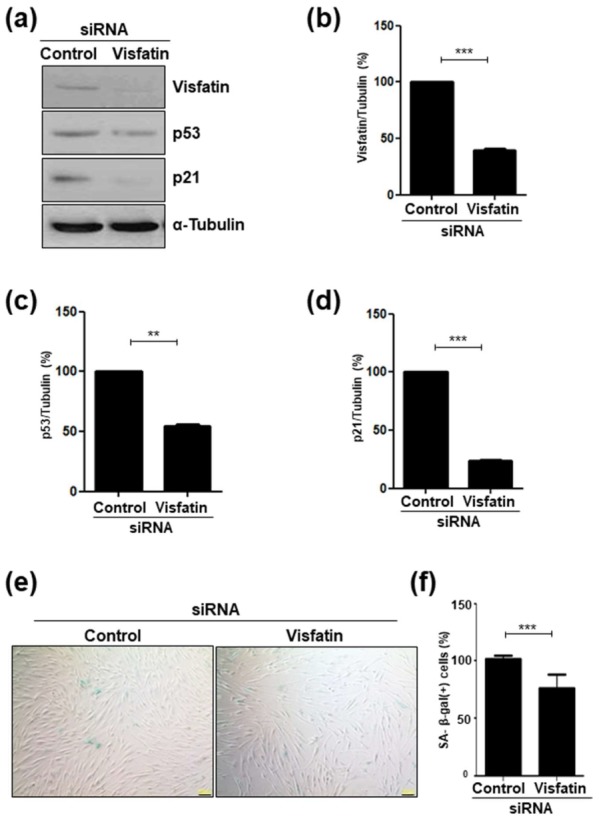
The knockdown of visfatin expression attenuates the senescence of human dental pulp cells (hDPCs). hDPCs were transfected with control siRNA or with visfatin siRNA for 48 h. (**a**) Transfected cell lysates were subjected to Western blotting to detect the levels of visfatin, p53, and p21 proteins. α-Tubulin was used as the loading control. (**b**–**d**) Densitometric analysis for assessing relative protein levels normalized with the levels of α-Tubulin protein: (**b**), visfatin; (**c**), p21; (**d**), p53. (**e**) Transfected cells were stained for detecting the activity of senescence-associated (SA)-β-galactosidase. Scale bar: 200 μm. (**f**) Quantification of the percentage of SA-β-galactosidase positively stained cells. ** *p* < 0.01, *** *p* < 0.001.

To evaluate whether visfatin is causally involved in the senescence of dental pulp cells, siRNA was used to knockdown visfatin expression. The transfection of cells with visfatin siRNA reduced visfatin protein levels (Figure 3a,b) as well as levels of p53 (Figure 3a,c) and p21 proteins (Figure 3a,d), which are leading aging markers. The SA-β-galactosidase staining assay showed a reduction in the fraction of SA-β-gal (+) cells in visfatin siRNA-transfected cells (Figure 3e,f).

### 3.4. Visfatin Treatment Accelerates Cellular Senescence

**Figure 4:**
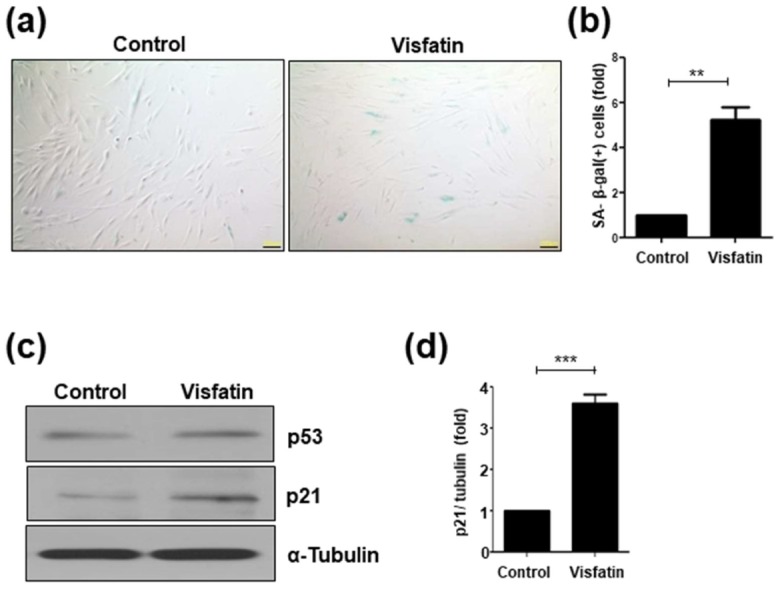
Exogenous visfatin treatment increases the senescence of human dental pulp cells (hDPCs). hDPCs were incubated with visfatin (500 ng/mL) for 24 h. (**a**) Representative image of senescence-associated (SA)-β-galactosidase staining. Scale bar: 200 μm. (**b**) Quantification of the percentage of SA-β-galactosidase positively stained cells. (**c**) Western blot analysis for detecting p53 and p21 proteins. α-Tubulin was used as the loading control. (**d**) Densitometric analysis for assessing relative p21 protein levels normalized to α-Tubulin protein levels. ** *p* < 0.01, *** *p* < 0.001.

To complement the results obtained using visfatin siRNA, whether treatment with exogenous visfatin induces cellular senescence was investigated. hDPCs were treated for 24 h with recombinant visfatin protein, then SA-β-galactosidase staining was assayed. Following visfatin treatment, the fraction of cells stained positive for SA-β-gal activity increased approximately 6-fold compared to that in the control (Figure 4a,b), which was confirmed by the detection of p53 and p21 expression. Although p53 protein levels were slightly increased (Figure 4c), the levels of p21 were significantly higher in visfatin-treated cells compared to control cells (Figure 4c,d).

### 3.5. FK866 Suppresses Senescence Induced by Visfatin

**Figure 5:**
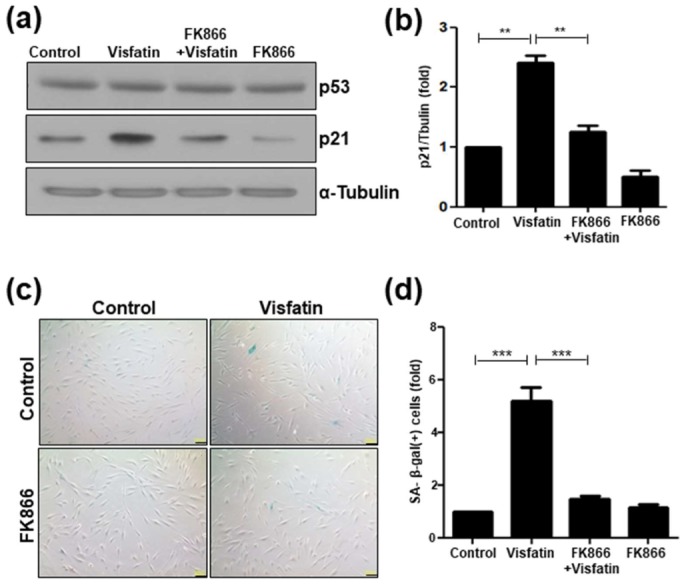
FK866 impedes the visfatin-induced senescence of human dental pulp cells (hDPCs). hDPCs were pretreated with FK866 (10 µM) for 2 h and then incubated with visfatin (500 ng/mL) for 24 h. (**a**) Cell extracts were subjected to Western blotting to detect p53 and p21 proteins. α-Tubulin was used as the loading control. (**b**) Densitometric analysis for assessing relative p21 protein levels were normalized to α-Tubulin protein levels. (**c**) Representative image of senescence-associated (SA)-β-galactosidase staining. Scale bar: 200 μm. (**d**) Quantification of the percentage of SA-β-galactosidase-positive cells. ** *p* < 0.01, *** *p* < 0.001.

To further examine the role of visfatin in cellular senescence, hDPCs were pretreated with FK866, a chemical inhibitor of visfatin/Nampt activity [34], prior to visfatin treatment. As shown in Figure 5a, p53 protein levels did not change significantly. In contrast, the visfatin-induced increase in p21 protein levels in hDPCs was significantly attenuated by pretreatment with FK866 (Figure 5a,b). Consistent with the effect on the levels of the senescence marker protein p21, the visfatin-induced increase in SA-β-gal(+) cells was reversed by FK866 treatment (Figure 5c,d).

### 3.6. Telomere Damage Mediates Visfatin-Induced Cellular Senescence

**Figure 6:**
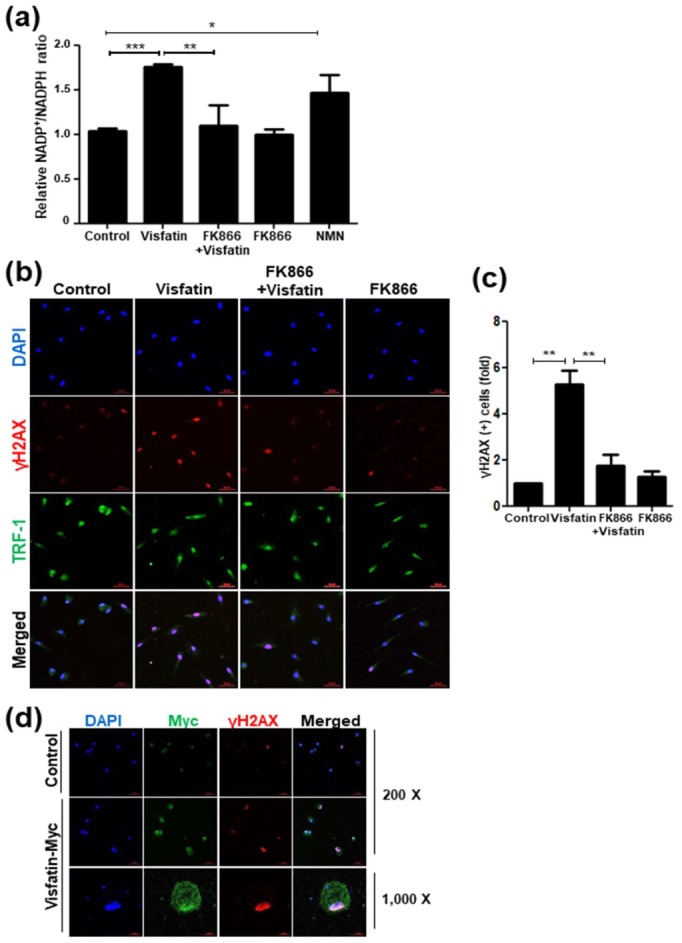
Visfatin increases NADPH consumption and induces telomere damage. Human dental pulp cells (hDPCs) were pretreated with FK866 (10 µM) for 2 h and then incubated with visfatin (500 ng/mL) for 24 h. (**a**) Measurement of the NADP/NADPH ratio in visfatin-treated hDPCs with or without FK866 pre-treatment. Nicotinamide mononucleotide (NMN, 100 µM), the product of the visfatin/Nampt enzymatic reaction, was used to compare its activity in NADPH consumption. (**b**) Immunofluorescence analysis of TRF-1 (Alexa fluoro (AF)-488, green) and γH2AX (AF-594, red) in hDPCs. The cells were analyzed using a confocal microscope. Nuclei were counterstained with DAPI (blue). Scale bar: 50 μm. (**c**) Quantification of the percentage of γH2AX-positive cells. (**d**) hDPCs were transiently transfected with a vector mediating the overexpression of visfatin (pCI-Visfatin-Myc) for 48 h and subjected to immunocytochemical analysis. Cells were stained using anti-Myc antibody (green) and anti-γH2AX (red). Nuclei were counterstained with DAPI (blue). Scale bar: 50 μm. * *p* < 0.1, ** *p* < 0.01, *** *p* < 0.001.

Cells are continuously exposed to various DNA damage-inducing stresses, such as oxidative stress [35]. Unless such DNA damage is thoroughly repaired, cells undergo premature senescence [36,37]. To examine the possibility that visfatin causes oxidative stress in dental pulp cells, NADP+/NADPH ratio, a major indicator of oxidative stress [38], was measured. It was found that visfatin increased NADP+/NADPH ratio, which was prevented by FK866 (Figure 6a). In addition, the stimulatory effect of visfatin on NADPH consumption was verified by using the product of the visfatin/Nampt enzymatic reaction, nicotinamide mononucleotide (NMN), as a positive control (Figure 6a).

Oxidative stress induces DNA damage, which is a major trigger of senescence [36]. To examine whether visfatin influences DNA damage response in dental pulp cells, immunocytochemistry was performed to detect γH2AX foci that are formed at sites of damaged DNA and uncapped dysfunctional telomeres [30,39,40]. The immunofluorescence intensity of γH2AX staining increased in response to visfatin (Figure 6b). In addition, visfatin caused an increase in the number of γH2AX foci, which were found scattered throughout the nucleus and colocalized with telomeric TRF1 signals (Figure 6b,c). The involvement of Nampt activity in the visfatin-induced DNA damage response was further examined. FK866 pretreatment caused a significant decrease in both the intensity and number of γH2AX(+) cells following visfatin treatment, yielding only basal levels of a DNA damage marker (Figure 6b,c).

To examine whether visfatin overexpression directly marks DNA-damaged cells, hDPCs were transfected with a visfatin overexpression vector (pCI-Visfatin-Myc), and immunostaining was performed using both anti-Myc and anti-γH2AX antibodies. The γH2AX-positive signals were stronger in the visfatin-overexpressed cells than in the control cells (Figure 6d, 200 X). In addition, when examined under high magnification (1000×), Myc-positive signals representing the overexpression of exogenous visfatin protein colocalized with γH2AX staining (Figure 6d), which is in line with a previous study that reported that visfatin induces telomere damage in endothelial cells [30].

### 3.7. Visfatin Upregulates the Expression of SASP Factors and Activates NF-κB p65

**Figure 7:**
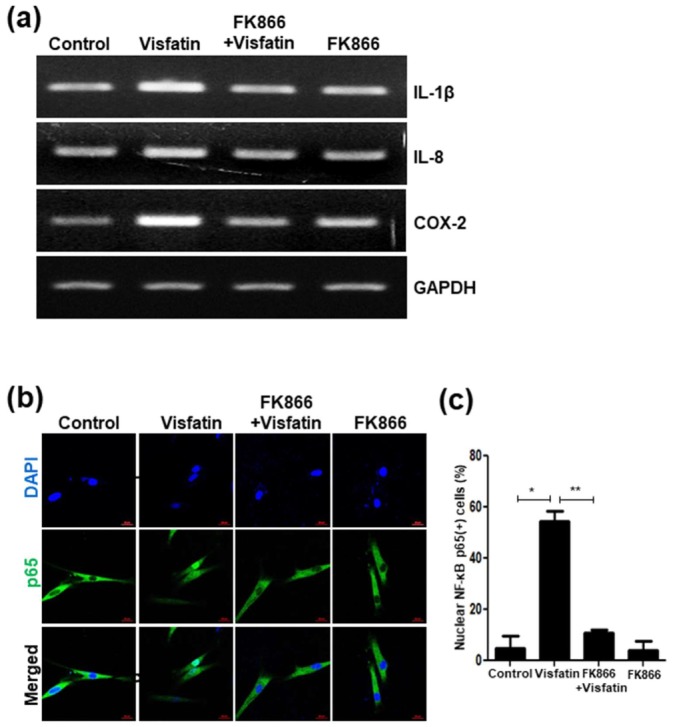
Increased levels of senescence-associated secretory phenotype (SASP) markers and activation of NF-κB p65 in hDPCs after exposure to visfatin. Human dental pulp cells (hDPCs) were pretreated with FK866 (10 µM) for 2 h and then incubated with visfatin (500 ng/mL) for 24 h. (**a**) Cell lysates were subjected to RT-PCR to determine the mRNA levels of indicated SASP markers. GAPDH was used as an internal control. (**b**) The immunocytochemical analysis of NF-κB p65 protein localization in visfatin-treated cells. Cells were evaluated using a fluorescence microscope. Nuclei were counterstained with DAPI (blue). (**c**) Quantification of the percentage of nuclear localization of NF-κB p65. Scale bar: 50 μm. * *p* < 0.1, ** *p* < 0.01.

The senescence-associated secretory phenotype (SASP) is a hallmark of senescent cells [41]. To investigate whether visfatin induces SASP, the expression levels of several SASP factors were examined by RT-PCR in visfatin-treated hDPCs. Visfatin increased the mRNA levels of SASP factors, such as IL-1b, IL-8, and COX-2, which were all reduced by the pretreatment of hDPCs with FK866 (Figure 7a). To determine whether the visfatin-induced upregulation in these SASP genes is related to NF-κB activation, which has been revealed to stimulate the transcription of many SASP genes, immunocytochemistry was performed. Visfatin significantly increases nuclear translocation of the p65 subunit of NF-κB, which was abrogated by FK866 pretreatment (Figure 7b,c).

## 4. Discussion

Obesity increases systemic inflammation and oxidative stress, and also affects cellular senescence and aging [42,43]. Adipocytokines, including adiponectin and visfatin, have previously been implicated in aging, longevity, and age-related diseases [44,45]. Adiponectin is secreted from adipose tissue and circulates as a hormone in the blood. It is decreased by various pathological conditions, such as obesity, diabetes, and coronary artery disease, and shows a negative correlation with age-related metabolic disturbances and a positive correlation with longevity [44]. While visfatin is highly expressed in cardiovascular and inflammatory diseases, as well as in various cancers [16], its role in tissue aging and cellular senescence is controversial. Visfatin has been shown to delay cellular senescence via NAD(+)-Sirt1 signaling in mesenchymal stem cells, endothelial progenitor cells, aortic smooth muscle cells, and fibroblasts [29,45,46]. In addition, inhibition of visfatin with FK866 has been shown to promote cellular senescence in retinal pigment epithelium [28]. In contrast, visfatin has been proposed as a positive factor for the induction of senescence in human endothelial cells [30]. The results of the present study show that visfatin induces senescence in hDPCs by stimulating NADPH consumption and thereby elevating oxidative stress. In addition, visfatin caused an increase in γH2AX foci in hDPCs, whereas FK866 attenuated visfatin-induced cellular senescence, which was accompanied by a decrease in γH2AX foci and the downregulation of p21. These results suggest that visfatin signaling induces dental pulp cell senescence, which is consistent with the results on human endothelial cells [30] but inconsistent with the inhibitory effects of visfatin on the senescence of other cell types [29,45,46]. The reason for this discrepancy is unclear but may result from cell-context-dependent visfatin signaling. Additional studies are needed to elucidate the mechanisms involved in the different effects of visfatin on cellular senescence.

Hallmarks of senescent cells include irreversible growth arrest and acquisition of a senescence-associated secretory phenotype (SASP) [36,41]. The SASP consists of a myriad of secreted factors, such as inflammatory cytokines, chemokines, growth factors, and matrix metalloproteases, that modulate tissue remodeling and tumor microenvironments and stimulate inflammation [36,41,47]. While the short-term induction of SASP can exert beneficial effects on tissue regeneration and tumor suppression through the clearance of damaged cells via auto/paracrine mechanisms, chronic exposure to SASP factors, such as in the case of aging or lesions, promotes inflammation and tumorigenesis as well as tissue aging and age-associated diseases [41,47,48]. Therefore, it is important to clarify the mechanism(s) responsible for the regulation of SASP. A recent study demonstrated that during senescence, high mobility group A (HMGA) upregulates visfatin/Nampt expression, which contributes to NF-κB activation via NAD(+) production [49]. The HMGA-NAMP-NAD(+)-NF-κB signaling axis initiates the expression of SASP factors with pro-inflammatory and tumor-promoting effects. These findings are consistent with our results indicating that visfatin upregulates the expression of SASP factors (IL-1β, IL-8, and COX-2) during dental pulp cell senescence and that NF-κB signaling activated by visfatin is involved, at least in part, in the senescence of dental pulp cells. Therefore, our data indicate that in terms of dental pulp cell senescence, both oxidative stress-induced DNA damage and SASP factors are major effectors in response to visfatin signaling, which chronically creates pro-inflammatory and pro-tumorigenic microenvironments, thereby contributing to inflammatory diseases, including tumorigenesis. In addition, since Nampt activity is involved in visfatin-induced senescence, FK866 can be considered an effective inhibitor of visfatin-induced senescence in dental pulp cells. Indeed, FK866, with anti-inflammatory and anti-tumorigenic activities, is already being tested in clinical trials [50].

To our knowledge, this is the first report on visfatin-induced senescence in association with telomere damage and the upregulation of SASP factors in human dental pulp cells. Considering the inductive roles of visfatin in dental pulp cell senescence, it is conceivable that the accumulation of senescent cells and the prolonged secretion of SASP factors by visfatin could further promote cellular senescence and inflammation in the dental pulp tissue, leading to aging-related diseases, including fibrosis and pulpitis. Therefore, additional in vivo studies are needed to examine the potential of visfatin as a therapeutic target for aging-related diseases of dental pulp tissues and to determine whether the visfatin inhibitor FK866 can be applied to maintain healthy dental pulp.

## Figures and Tables

**Table 1:** Clinical information of patient samples analyzed for immunohistochemical expression of visfatin in dental pulp tissues of subjects with young group and old group.

Age		No. of Patients	Gender	
			Male	Female
Young	~19	4	4	0
	~29	3	2	1
Old	~39	3	0	3
	~49	3	2	1
Total		13	8	5
